# The BARD1 Cys557Ser Variant and Breast Cancer Risk in Iceland

**DOI:** 10.1371/journal.pmed.0030217

**Published:** 2006-06-20

**Authors:** Simon N Stacey, Patrick Sulem, Oskar T Johannsson, Agnar Helgason, Julius Gudmundsson, Jelena P Kostic, Kristleifur Kristjansson, Thora Jonsdottir, Helgi Sigurdsson, Jon Hrafnkelsson, Jakob Johannsson, Thorarinn Sveinsson, Gardar Myrdal, Hlynur Niels Grimsson, Jon T Bergthorsson, Laufey T Amundadottir, Jeffrey R Gulcher, Unnur Thorsteinsdottir, Augustine Kong, Kari Stefansson

**Affiliations:** 1deCODE Genetics, Reykjavik, Iceland; **2**Department of Oncology, National University Hospital, Reykjavik, Iceland; **3**Cancer Centre, National University Hospital, Reykjavik, Iceland; **4**Department of Radiation Physics, National University Hospital, Reykjavik, Iceland; Guy's King's and St Thomas' School of MedicineUnited Kingdom

## Abstract

**Background:**

Most, if not all, of the cellular functions of the BRCA1 protein are mediated through heterodimeric complexes composed of BRCA1 and a related protein, BARD1. Some breast-cancer-associated BRCA1 missense mutations disrupt the function of the BRCA1/BARD1 complex. It is therefore pertinent to determine whether variants of BARD1 confer susceptibility to breast cancer. Recently, a missense BARD1 variant, Cys557Ser, was reported to be at increased frequencies in breast cancer families. We investigated the role of the BARD1 Cys557Ser variant in a population-based cohort of 1,090 Icelandic patients with invasive breast cancer and 703 controls. We then used a computerized genealogy of the Icelandic population to study the relationships between the Cys557Ser variant and familial clustering of breast cancer.

**Methods and Findings:**

The Cys557Ser allele was present at a frequency of 0.028 in patients with invasive breast cancer and 0.016 in controls (odds ratio [OR] = 1.82, 95% confidence interval [CI] 1.11–3.01, *p =* 0.014). The alleleic frequency was 0.037 in a high-predisposition group of cases defined by having a family history of breast cancer, early onset of breast cancer, or multiple primary breast cancers (OR = 2.41, 95% CI 1.22–4.75, *p =* 0.015). Carriers of the common Icelandic BRCA2 999del5 mutation were found to have their risk of breast cancer further increased if they also carried the BARD1 variant: the frequency of the BARD1 variant allele was 0.047 (OR = 3.11, 95% CI 1.16–8.40, *p =* 0.046) in 999del5 carriers with breast cancer. This suggests that the lifetime probability of a BARD1 Cys557Ser/BRCA2 999del5 double carrier developing breast cancer could approach certainty. Cys557Ser carriers, with or without the BRCA2 mutation, had an increased risk of subsequent primary breast tumors after the first breast cancer diagnosis compared to non-carriers. Lobular and medullary breast carcinomas were overrepresented amongst Cys557Ser carriers. We found that an excess of ancestors of contemporary carriers lived in a single county in the southeast of Iceland and that all carriers shared a SNP haplotype, which is suggestive of a founder event. Cys557Ser was found on the same SNP haplotype background in the HapMap Project CEPH sample of Utah residents.

**Conclusions:**

Our findings suggest that BARD1 Cys557Ser is an ancient variant that confers risk of single and multiple primary breast cancers, and this risk extends to carriers of the BRCA2 999del5 mutation.

## Editors' Summary

**Background.:**

About 13% of women (one in eight women) will develop breast cancer during their lifetime, but many factors affect the likelihood of any individual woman developing this disease, for example, whether she has had children and at what age, when she started and stopped her periods, and her exposure to certain chemicals or radiation. She may also have inherited a defective gene that affects her risk of developing breast cancer. Some 5%–10% of all breast cancers are familial, or inherited. In 20% of these cases, the gene that is defective is *BRCA1* or *BRCA2*. Inheriting a defective copy of one of these genes greatly increases a woman's risk of developing breast cancer, while researchers think that the other inherited genes that predispose to breast cancer—most of which have not been identified yet—have a much weaker effect. These are described as low-penetrance genes. Inheriting one such gene only slightly increases breast cancer risk; a woman has to inherit several to increase her lifetime risk of cancer significantly.

**Why Was This Study Done?:**

It is important to identify these additional predisposing gene variants because they might provide insights into why breast cancer develops, how to prevent it, and how to treat it. To find low-penetrance genes, researchers do case–control association studies. They find a large group of women with breast cancer (cases) and a similar group of women without cancer (controls), and examine how often a specific gene variant occurs in the two groups. If the variant is found more often in the cases than in the controls, it might be a variant that increases a woman's risk of developing breast cancer.

**What Did the Researchers Do and Find?:**

The researchers involved in this study recruited Icelandic women who had had breast cancer and unaffected women, and looked for a specific variant—the Cys557Ser allele—of a gene called *BARD1*. They chose *BARD1* because the protein it encodes interacts with the protein encoded by *BRCA1*. Because defects in *BRCA1* increase the risk of breast cancer, defects in an interacting protein might have a similar effect. In addition, the Cys557Ser allele has been implicated in breast cancer in other studies. The researchers found that the Cys557Ser allele was nearly twice as common in women with breast cancer as in control women. It was also more common (but not by much) in women who had a family history of breast cancer or who had developed breast cancer more than once. And having the Cys557Ser allele seemed to increase the already high risk of breast cancer in women who had a *BRCA2* variant (known as *BRCA2* 999del5) that accounts for 40% of inherited breast cancer risk in Iceland.

**What Do These Findings Mean?:**

These results indicate that inheriting the *BARD1* Cys557Ser allele increases a woman's breast cancer risk but that she is unlikely to have a family history of the disease. Because carrying the Cys557Ser allele only slightly increases a woman's risk of breast cancer, for most women there is no clinical reason to test for this variant. Eventually, when all the low-penetrance genes that contribute to breast cancer risk have been identified, it might be helpful to screen women for the full set to determine whether they are at high risk of developing breast cancer. This will not happen for many years, however, since there might be tens or hundreds of these genes. For women who carry *BRCA2* 999del5, the situation might be different. It might be worth testing these women for the *BARD1* Cys557Ser allele, the researchers explain, because the lifetime probability of developing breast cancer in women carrying both variants might approach 100%. This finding has clinical implications in terms of counseling and monitoring, as does the observation that Cys557Ser carriers have an increased risk of a second, independent breast cancer compared to non-carriers. However, all these findings need to be confirmed in other groups of patients before anyone is routinely tested for the *BARD1* Cys557Ser allele.

**Additional Information.:**

Please access these Web sites via the online version of this summary at http://dx.doi.org/10.1371/journal.pmed.0030217.

• MedlinePlus pages about breast cancer

• Information on breast cancer from the United States National Cancer Institute

• Information on inherited breast cancer from the United States National Human Genome Research Institute

• United States National Cancer Institute information on genetic testing for BRCA1 and BRCA2 variants

• GeneTests pages on the involvement of BRCA1 and BRCA2 in hereditary breast and ovarian cancer

• Cancer Research UK's page on breast cancer statistics

## Abstract 3

In a population-based cohort of 1090 Icelandic patients, a Cys557Ser missense variant of the BARD1 gene, which interacts with BRCA1, increased the risk of single and multiple primary breast cancers.

## Introduction

Since the discovery of the BRCA1 and BRCA2 genes, much attention has been focused on characterizing the remaining genetic risk of breast cancer. It is typically estimated that strongly predisposing mutations in BRCA1 and BRCA2 account for 15%–25% of the familial component of the risk [ 1, 2]. Data from twin studies and studies of the high incidence of cancer in the contralateral breast of patients surviving primary breast cancer suggest that a substantial portion of the uncharacterized risk of breast cancer is genetic, even in the absence of a strong family history of the disease [ 3, 4]. Model-fitting studies have indicated that the residual genetic risk is likely to be polygenic in nature [ 5– 7]. This predicted genetic heterogeneity, together with the rather limited success of family-based linkage studies, has led to a shift in focus towards a search for genes with variants that are less penetrant.

We and others have shown that there is a significant familial risk of breast cancer in Iceland that extends to at least fifth-degree relatives [ 8, 9]. The contribution of BRCA1 mutations to familial risk in Iceland is thought to be minimal [ 10, 11]. A single founder mutation in the BRCA2 gene (999del5) is present at a carrier frequency of 0.6%–0.8% in the general Icelandic population and 7.7%–8.6% in female breast cancer patients [ 12, 13]. This single mutation is estimated to account for approximately 40% of the inherited breast cancer risk for first- through third-degree relatives [ 9]. Although this estimate is higher than the 15%–25% of familial risk attributed to all BRCA1 and BRCA2 mutations combined in non-founder populations, there is still some 60% of Icelandic familial breast cancer risk to be explained. First-degree relatives of breast cancer patients who test negative for BRCA2 999del5 remain at a 1.72-fold higher risk for breast cancer than the overall population (95% CI 1.49–1.96) [ 9]. The understanding of the genetic factors contributing to this residual risk is very limited.

The majority of the BRCA1 protein in vivo exists as heterodimeric complexes with BARD1, an interaction mediated through related RING finger domains present in both proteins. The complex is important for the roles of BRCA1 in homologous-recombination-directed DNA repair and transcription-coupled repair [ 14, 15]. The integrity of the BRCA1/BARD1 complex is crucial for normal development, as both BRCA1 and BARD1 knockout mice and frogs die as embryos [ 16, 17]. In most tissues, expression of BRCA1 and BARD1 is regulated in a coordinated fashion [ 18]. Under- or overexpression of either component can lead to apoptosis, suggesting that an unbalanced expression or a disruption of the complex activates pro-apoptotic effector functions [ 19– 21].

The importance of the integrity of BRCA1/BARD1 complexes is further underlined by the finding in breast cancer families of missense mutations in the BRCA1 RING finger domain. The common pathogenic substitutions C61G and C64G occur in the zinc-binding residues of the BRCA1 RING finger domain, disrupting its structure and abolishing its E3 ubiquitin ligase activity [ 22, 23]. A relevant question is whether mutations or variants in the BARD1 gene also associate with breast cancer risk. Occasional reports have appeared describing BARD1 variants in isolated cancer families or as low-frequency population variants [ 24– 27]. Recently, attention has focused on the Cys557Ser variant. Cys557 occurs between the ankyrin repeats and BRCT domains present on the BARD1 protein. This region has been implicated in pro-apoptotic effector functions and inhibition of the mRNA 3′ end processing factor CstF1 [ 28– 30]. Ectopically expressed Cys557Ser protein shows defects in growth-suppressive and pro-apoptotic functions, suggesting that the variant may be pathogenic [ 31].

The Cys557Ser variant was first reported with a carrier frequency of about 4% in a normal population with European ancestry [ 27]. Subsequently it was observed in an Italian family with cases of breast and ovarian cancer, but was absent from a control sample of 60 individuals without breast or ovarian cancer [ 24]; recently, the Cys557Ser variant was found at a frequency of 5.6% in Finnish families with a history of breast and ovarian cancer and at a frequency of 7.4% in families where breast cancer without ovarian cancer was prevalent [ 26]. In this study, we sought to illuminate the role of the BARD1 Cys557Ser variant in breast cancer using a population-based case–control set representing all consenting patients who were diagnosed with breast cancer in Iceland between 1955 and 2004.

## Materials

### Patient and Control Selection

Approval for the study was granted by the National Bioethics Committee of Iceland and the Icelandic Data Protection Authority. Records of breast cancer diagnoses were obtained from the Icelandic Cancer Registry (ICR) of the Icelandic Cancer Society. The records included all cases of invasive breast tumors and ductal or lobular carcinoma in situ diagnosed in Iceland from 1 January 1955 to 31 March 2004. Ductal and lobular carcinoma in situ have been recorded since 1955; however, in practice very few cases were diagnosed prior to the initiation of the national breast screening program in November 1987. There were 4,585 diagnoses in 4,306 individuals during this period. Of these, 4,255 diagnoses were invasive cancer and 330 were ductal or lobular carcinoma in situ. For analyses of cancer risk and age of onset, only International Classification of Diseases code 10 cases for invasive breast cancer in females were used. In familial clustering analyses, in situ carcinomas and male breast cancers were included. In situ carcinomas were also considered as second primary tumors. ICR records were histologically verified in over 95% of the cases. For analysis of morphological subtypes, only histologically verified material was used. Second primary tumors were confirmed both clinically and by histology to be independent primary tumors, arising simultaneously or subsequently to the first breast cancer and occurring in the contralateral or ipsilateral breast. In analysis of second primary tumors, all diagnoses of new independent primaries were considered, so an individual could have more than two tumors diagnosed. All living patients with a diagnosis in the ICR were eligible for participation in the study. Recruitment took place between September 2003 and April 2005. During the recruitment period, a total of 1,997 patients were alive and eligible to participate. We were able to contact 1,926 (96%) of these patients, and 1,431 (74% of those contacted) consented to participate. Of the consenting patients, 1,241 (87%) were successfully genotyped for the BARD1 variant. Patients were asked to identify close relatives who could be invited to participate in the study. In this study, genotypic data from relatives were used only to provide phase information for BARD1 Cys557Ser-variant-associated single nucleotide polymorphism (SNP) haplotypes and for inheritance error checking of the patients' genotypes.

In order to generate the control group, we selected randomly from the Icelandic genealogical database 1,034 triads (two parents and one offspring) with all individuals between the ages of 18 and 70 y. The selected individuals were invited to participate in the study, and 1,023 were recruited. The medical histories of the controls were not investigated. Genotyping was carried out for BARD1 Cys557Ser and BRCA2 999del5. From among the successfully genotyped individuals, only those who were unrelated to each other at a distance of at least three meiotic events were selected for use as controls. Where complete triads were available, the parental component was used. This resulted in a group of 703 genotyped, unrelated individuals with a median age of 53 y and a sex ratio of 1.0. There was no difference in the carrier frequencies of the BARD1 Cys557Ser variant between males and females in the control group ( *p =* 0.40). Genotyped offspring from triads, where available, were used to establish phase information for the BARD1 Cys557Ser-variant-associated SNP haplotypes and for error checking of the controls' genotypes. However, the offspring were not counted as controls.

HapMap Project samples consisted of 30 triads from the Centre d'Etude du Polymorphisme Humain (CEPH) population (Utah, United States, residents with ancestry from northern and western Europe); 45 unrelated Han Chinese individuals from Bejing, China; 45 unrelated Japanese individuals from Tokyo, Japan; and 30 triads from the Yoruba population in Ibadan, Nigeria. Samples were obtained as lymphoblastoid cell lines from the Coriell Institute for Medical Research (Camden, New Jersey, United States).

### Genotyping

All personal identifiers on samples, pedigrees, and medical information were encrypted by representatives of the Icelandic Data Protection Authority prior to entry into the study [ 32]. Blood samples were preserved in EDTA at −20 °C. DNA was isolated from whole blood or lymphoblastoid cell lines using a Qiagen ( http://www.qiagen.com) extraction column method. Cys557Ser typing was carried out by DNA sequencing of BARD1 exon 7. Exon 6 was also sequenced in order to read the genotypes of a number of public domain SNPs in this exon. PCR amplifications and sequencing reactions were set up on Zymark ( http://www.zymark.com) SciClone ALH300 robotic workstations and amplified on MJR ( http://www.mjr.com) Tetrads. PCR products were verified for correct length by agarose gel electrophoresis and purified using AMPure (Agencourt Bioscience; http://www.agencourt.com). Purified products were sequenced using an ABI PRISM Fluorescent Dye Terminator system (PerkinElmer; http://www.perkinelmer.com), repurified using CleanSEQ (Agencourt), and resolved on Applied Biosystems 3730 capillary sequencers. SNP calling from primary sequence data was carried out using deCODE Genetics ( http://www.decode.com) Clinical Genome Miner software. BRCA2 999del5 mutations were detected using a microsatellite-type PCR assay. All BARD1 Cys557Ser and BRCA2 999del5 variants identified by the automated systems were confirmed by manual inspection of primary signal traces. Phase information for SNP haplotypes was revealed by genotyping patients' family members and by genotyping triads from control and HapMap Project samples. Phase and haplotype frequencies were determined using deCODE Genetics Allegro and NEMO software [ 33, 34].

### Genealogical Database

deCODE Genetics maintains a computerized database of the genealogy of Icelanders. The records include almost all individuals born in Iceland in the last two centuries, and for that period around 95% of the parental connections are known [ 35]. In addition, a county-of-residence identifier is recorded for most individuals, based on census and parish records. The information is stored in a relational database with encrypted personal identifiers that match those used on the biological samples and ICR records, allowing cross-referencing of the genotypes and phenotypes of the study participants with their genealogies.

### Statistical Methods

We calculate the odds ratio (OR) of the frequency of BARD1 Cys557Ser as



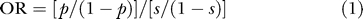



where *p* and *s* are the frequencies of Cys557Ser in the patients and the controls, respectively. Because the frequency of Cys557Ser is low, ORs for allele frequencies are very similar to ORs for carrier status in patients and controls. With population controls, it can be shown through Bayes's Rule that the OR as defined above, and calculated for all breast cancer patients, corresponds to Risk(carrier)/Risk(non-carrier) where Risk is the probability of breast cancer given carrier status. *p*-Values associated with ORs were calculated based on a standard likelihood ratio Chi-square statistic. Confidence intervals were calculated assuming that the estimate of OR has a log-normal distribution.

When OR is calculated using breast cancer patients who are also carriers of BRCA2 999del5 compared to population controls, OR is an estimate of the risk ratio of BRCA2 999del5 carriers who are also carriers of BARD1 Cys557Ser compared to BRCA2 999del5 carriers who are not carriers of BARD1 Cys557Ser. This is because, by applying Bayes's Rule and assuming that BARD1 and BRCA2 are in linkage equilibrium in the general population, it can be shown that



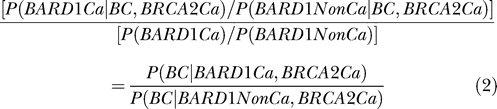



where *BC* denotes breast cancer, and *Ca* and *NonCa* denote variant carrier and non-carrier, respectively. In other words, when the OR is greater than one, it indicates that the risk for BRCA2 999del5 carriers is further increased if they also carry BARD1 Cys557Ser.

The 10-y risk for multiple primary tumors was determined for each genotype class as (the number of secondary diagnostic events/number of person-years of follow-up) × 10. The total number of person-years of follow-up available for analysis was 554 for BARD1 Cys557Ser carriers, 12,136 for non-carriers of Cys557Ser, 492 for BARD1 Cys557Ser carriers who were proven BRCA2 999del5 non-carriers, 11,371 for non-carriers of both BARD1 Cys557Ser and BRCA2 999del5, 817 for BRCA2 999del5 carriers, and 13,675 for 999del5 non-carriers. Because it was possible for individuals to be diagnosed with more than one additional primary tumor after the first diagnosis, we employed a randomization simulation strategy to determine the significance of the frequencies of subsequent primary diagnoses. Significance was assessed by 10,000 simulations in which carrier status was assigned randomly among the tested individuals, and the frequency of subsequent primary diagnoses was determined for each simulation. An empirical *p*-value was then assigned to the observed frequency of subsequent primary diagnoses in carriers by reference to the distribution derived from the simulations. Histological subclasses were analyzed by multivariate analysis with age taken into account as a continuous covariate. In order to assess the significance of geographical distributions of ancestors of Cys557Ser carriers, 1,000 lists of random age- and sex-matched controls were generated from the genealogical database. The geographical ancestry for each list was traced back five generations, providing a null distribution against which the observed distribution of geographical ancestry for Cys557Ser carriers could be compared. This allowed the statistical significance of geographical clustering in ancestry to be evaluated for each county using an empirical *p*-value. Bonferroni correction was applied in order to correct for the number of counties evaluated. Age of onset comparisons were assessed by Wilcoxon tests run on JMP version 4 software (SAS Institute; http://www.sas.com). All *p*-values are reported as two-sided.

## Results

### The Frequency of the BARD1 Cys557Ser Variant Is Increased in Icelandic Breast Cancer Cases

Breast cancer cases diagnosed in Iceland from January 1955 to March 2004 were ascertained from ICR records. A total of 1,090 patients diagnosed with invasive breast cancer were successfully typed for the BARD1 Cys557Ser variant by DNA sequencing. Population-based controls were selected randomly from the national genealogical database. We used the genealogical database to identify a set of 992 genotyped patients and 703 controls who were unrelated to each other by a distance of three meiotic events, in order to control for the potential effect of relatedness among the groups. The patients showed a significantly greater frequency of the Cys557Ser allele than the controls (OR = 1.82, 95% CI 1.11–3.01, *p =* 0.014; Table 1), demonstrating that the BARD1 variant confers risk for breast cancer in Iceland. Fifty-two patients were heterozygous and two patients were homozygous for the Cys557Ser variant. The homozygosity was confirmed by analysis of six SNPs flanking the variant (see below). All controls who carried the variant were heterozygotes. Thus, the carrier frequency of Cys557Ser was 5.44% in cases and 3.13% in controls.

**Table 1:**
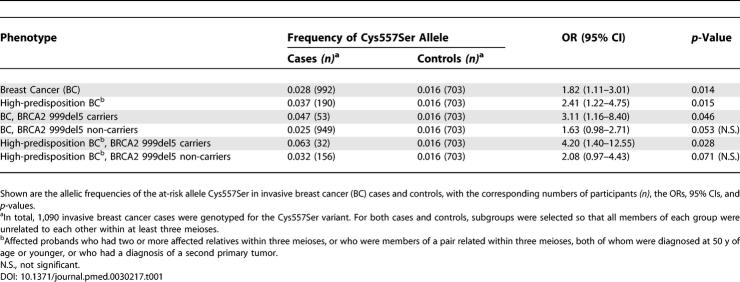
Association of the BARD1 Cys557Ser Allele with Breast Cancer in Iceland

Because the ICR records go back to 1955, some of the patients we recruited were long-term cancer survivors. If patients who carried Cys557Ser have different probabilities of long-term survival than non-carriers, then the frequency of the variant amongst prevalent cases might be affected. To investigate this, the analysis was repeated on a subset of patients composed of 310 unrelated individuals who were diagnosed after 1 January 2000, with times from diagnosis to recruitment of less than 5 y. In this cohort of recently diagnosed patients the frequency of the Cys557Ser allele was 0.031, again significantly greater than for controls (OR = 1.99, 95% CI 1.07–3.70, *p =* 0.033). We also compared this cohort of 310 recently diagnosed patients to a group of 389 unrelated patients who had been diagnosed before 1 January 1995 and had survived to recruitment. The frequency of the Cys557Ser allele in this group of long-term survivors was 0.026, which is not significantly different from that of the recently diagnosed patients ( *p =* 0.579). Therefore, there is no compelling evidence for an influence of Cys557Ser on patient survival.

In order to assess the role of the Cys557Ser allele in patients showing high predisposition to breast cancer, we identified a set of patients who had two or more affected relatives within three meiotic events, or who were members of a pair related within three meiotic events, both of whom were diagnosed at age 50 y or younger, or who had a recorded diagnosis of a second independent primary tumor. This set of patients was designated “high-predisposition breast cancer.” For each high-predisposition cluster identified, only a single representative was chosen for analysis at random from the genotyped individuals, resulting in a set of 190 independent high-predisposition probands. As shown in Table 1, the frequency of the Cys557Ser allele was greater in this high-predisposition group than in controls (OR = 2.41, 95% CI 1.22–4.75, *p =* 0.015). The point estimate of the OR was nominally (but not significantly) higher than that observed for patients who were not selected for high predisposition, as would be expected of a genetic factor contributing modestly to familial predisposition.

Since the BRCA2 999del5 mutation has such a substantial impact on familial breast cancer in Iceland, we considered its relationship with the BARD1 Cys557Ser variant. One possible scenario would be that the BARD1 Cys557Ser variant confers negligible additional risk to BRCA2 999del5 carriers, as has been suggested for the interaction between CHEK2 and BRCA mutations [ 36, 37]. If this were so, then the frequency of the BARD1 variant among affected BRCA2 999del5 carriers would approximate the control frequency. Conversely, if the frequency of the BARD1 variant in affected BRCA2 999del5 carriers is greater than in the population controls, it would imply that the BARD1 variant confers risk on the BRCA2 carriers over and above the risk conferred by the 999del5 mutation (see Materials for a more detailed discussion of this concept).

To investigate a potential risk from Cys557Ser in BRCA2 999del5 carriers, a set of unrelated 999del5 carriers was identified among the 1,090 patients typed for the Cys557Ser variant. The frequency of the Cys557Ser variant in 999del5 mutation carriers, both those not selected for family history and those selected for high predisposition, was significantly greater than in population controls (OR = 3.11, 95% CI 1.16–8.40, *p =* 0.046 for non-selected 999del5 carriers; OR = 4.20, 95% CI 1.40–12.55, *p =* 0.028 for 999del5 carriers selected for high-predisposition; Table 1). Therefore, BRCA2 999del5 carriers (who are already at high risk of breast cancer) have their risk multiplied by an estimated factor of 3.11 if they also carry the BARD1 Cys557Ser variant. These observations demonstrate that the increased risk of breast cancer conferred by the Cys557Ser variant extends to BRCA2 999del5 mutation carriers.

### BARD1 Cys557Ser and Familial Clustering of Breast Cancer

**Figure 1:**
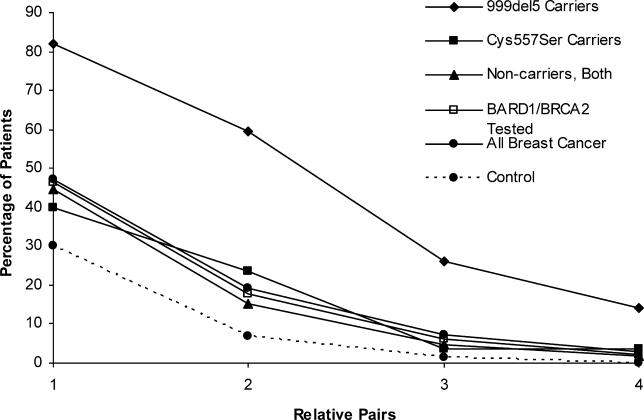
Proportions of BARD1 Cys557Ser Patients, BRCA2 999del5 Patients, and Reference Groups of Patients Showing Family Histories of Breast Tumors For each member of the group of Cys557Ser carrier patients ( *n =* 55), the genealogical database and ICR records of diagnoses were searched to identify all relatives with breast tumors within a distance of three meioses. The proportion of Cys557Ser carriers who had one or more affected relatives, two or more affected relatives, and so on is indicated. For comparison, the analysis was repeated for BRCA2 999del5 patients ( *n =* 84), non-carriers of both BARD1 and BRCA2 variants ( *n =* 1,091), all patients who were tested for both variants ( *n =* 1,209), and all patients in the ICR records ( *n =* 4,306). Controls were 703 individuals chosen randomly from the genealogical database.

It is well known that breast cancer tends to occur in familial clusters [ 8, 38]. The 1.82-fold increased risk of breast cancer conferred by the BARD1 Cys557Ser variant will, by definition, make a contribution towards familial clustering of affected carriers. The overall degree of familial clustering in affected Cys557Ser carriers will also depend on how the variant acts in combination with other predisposition genes and environmental factors shared within families. It is important to assess, therefore, what proportion of patients who carry the Cys557Ser variant have a family history of breast cancer. The availability of the Icelandic genealogical database, along with complete records of breast cancer diagnoses in Iceland since 1955, makes it possible to observe directly the tendencies of BARD1 Cys557Ser variant carriers to participate in familial clusters of breast cancer. Starting with the group of Cys557Ser patients, we queried the genealogy for what fraction of them were in one or more relative pairs, within a distance of three meioses, with other patients in the group of 4,306 patients in the ICR. In other words, we queried what proportion of the variant carrier patients had at least one first- or second-degree relative diagnosed with breast cancer. We then queried what proportion of Cys557Ser variant carriers had two or more, three or more, and four or more affected relatives within the same genetic distance ( Figure 1).

In order to put the analysis into context, we similarly tested the tendency of BRCA2 999del5 mutation carriers to participate in familial breast cancer clusters. We also tested the clustering driven by several reference groups of breast cancer patients: the 1,091 patients who were proven non-carriers of either Cys557Ser or 999del5, the 1,209 patients who had been tested for both Cys557Ser and 999del5 (regardless of the carrier status thereby identified), and the entire group of 4,306 patients in the ICR records. The tendency of the 703 control group members to participate in familial breast cancer clusters was also examined. When compared to the controls, the reference groups of breast cancer patients all had more affected relatives, as expected ( Figure 1). The patients carrying the Cys557Ser variant also had more affected relatives than the controls. They did not, however, exhibit a markedly greater tendency to participate in familial breast cancer clusters than the breast cancer reference groups ( Figure 1). Only the BRCA2 mutation carriers showed a distinctly stronger tendency to contribute to familial clusters than the reference groups. Therefore, unlike BRCA2 breast cancer patients, patients who carry the BARD1 variant do not present with family histories in a frequency that would distinguish them from the overall population of breast cancer patients.

### Age at Diagnosis of Breast Cancer in Patients Carrying the BARD1 Cys557Ser Variant

The median age at diagnosis of BARD1 Cys557Ser carrier breast cancer patients was 55.1 y. This is not significantly different from BARD1 non-carriers (median 55.9 y). The median age of breast cancer diagnosis of BRCA2 999del5 carriers was 48.1 y, significantly younger than non-carriers of the BRCA2 mutation ( *p* < 0.001). Patients carrying both BARD1 Cys557Ser and BRCA2 999del5 had a median age of onset of 44.1 y; however, this was not significantly different from 999del5-only carriers ( *p =* 0.498). The two patients who were shown to be homozygous for the Cys557Ser variant had quite early onset of the disease, at ages 41 and 47 y. Neither of the Cys557Ser homozygotes were 999del5 carriers.

### Increased Incidence of Multiple Primary Breast Tumors in BARD1 Cys557Ser Carriers

The occurrence of multiple primary tumors is an indication of hereditary breast cancer predisposition. Therefore, we determined whether multiple primary breast tumors (invasive or in situ) occurred at higher than expected frequencies in Cys557Ser carriers ( Table 2). The mean follow-up time (from diagnosis to recruitment) for the entire cohort was 10.3 y. We determined the 10-y risk for multiple primary tumors and compared risks of carriers and non-carriers to determine risk ratios. Significance was assessed by empirical *p*-values derived from 10,000 simulations of individuals with randomly assigned carrier status. As shown in Table 2, the risk of multiple primary tumors was more than doubled in BARD1 Cys557Ser carriers compared to non-carriers. Interestingly, the risk of multiple primary tumors was also increased among BARD1 Cys557Ser carriers who had tested negative for BRCA2 999del5 mutations, indicating that the effect of the BARD1 variant is not restricted to BRCA2 mutation carriers. The risk of second primary breast tumors was significantly greater in the group of all BRCA2 999del5 mutation carriers than in non-carriers, as expected.

**Table 2:**
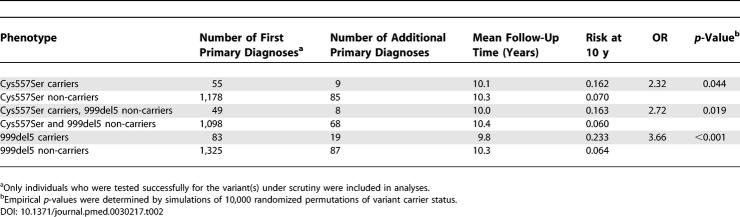
The Risk of Multiple Primary Tumors in BARD1 Cys557Ser and BRCA2 999del5 Carriers

### The BARD1 Cys557Ser Variant Is Overrepresented in Lobular and Medullary Carcinomas of the Breast

We next sought to determine whether the Cys557Ser variant associates preferentially with specific histological classes of breast cancer as defined by SNOMED morphology codes ( http://www.snomed.org). The most frequent histological class in both carriers and non-carriers was infiltrating ductal carcinoma, as expected ( Table 3). However, there was a significant difference in the distribution of the less common histological classes, with an approximate 2.5-fold excess of lobular carcinoma and 6.9-fold excess of medullary carcinoma among carriers of the Cys557Ser variant. Carcinomas in situ were absent from Cys557Ser carriers, whereas they were present at a frequency 12% in non-carriers (age-adjusted *p* < 0.001), suggesting more aggressiveness of BARD1 variant tumors. The analysis of histological classes was repeated excluding carcinoma in situ diagnoses, and showed a significant difference in distribution of the invasive histological types between carriers and non-carriers (age-adjusted *p =* 0.011).

**Table 3:**
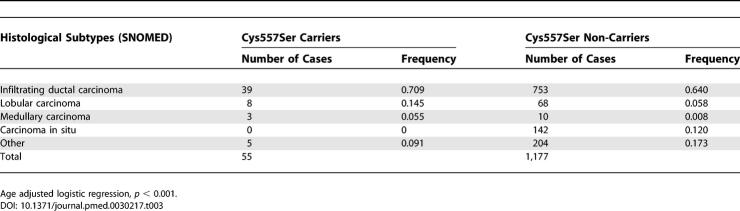
Distribution of Histological Subtypes of First Primary Breast Tumor Diagnoses in BARD1 Cys557Ser Carriers and Non-Carriers

### The Geographical Ancestry of the BARD1 Cys557Ser Variant Can Be Traced to a Single County in the South East of Iceland

Icelanders are now highly urbanized, but in the past regional subpopulations were more isolated [ 39]. By examining the geographical distribution of ancestors for a group of individuals carrying a given genetic variant, it is possible to shed light on the history of the variant. In particular, this approach can indicate the nature and extent of a possible founder effect in the history of the BARD1 Cys557Ser variant and whether the ancestors from whom modern Icelanders have inherited the variant lived predominantly in a particular region of Iceland.

**Figure 2:**
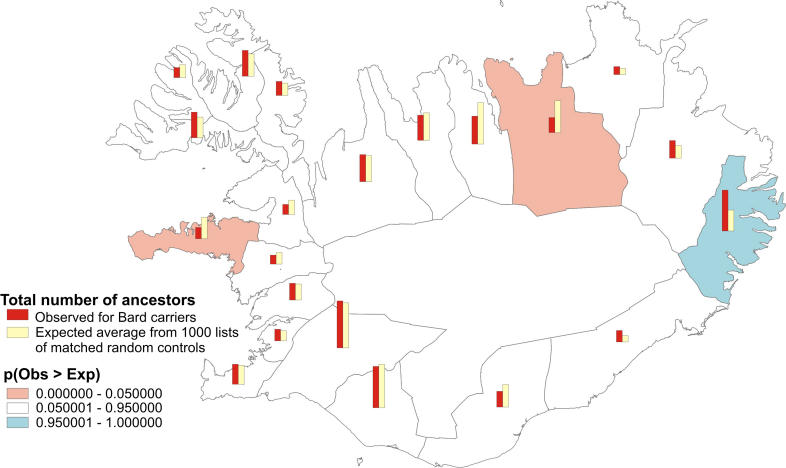
Geographical Ancestry of All Known BARD1 Cys557Ser Carriers Red bars indicate the locations of ancestors in the fifth generation back for Cys557Ser carriers, and yellow bars represent the average number of ancestors expected for each county based on 1,000 lists of randomly selected age- and sex-matched controls. One county in the east, S-Múlasýsla (shaded in blue), shows an excess of BARD1 Cys557Ser ancestors that retained significance after Bonferroni correction. Two counties (shaded in pink) exhibited a marginally significant deficit of BARD1 Cys557Ser ancestors, which did not survive Bonferroni correction.

For each Cys557Ser carrier identified in the study (irrespective of breast cancer affection status) all the known ancestors five generations back were identified, yielding a maximum number of 32 (2 ^5^) ancestors for each contemporary carrier. The proportions of ancestors originating from each county were then determined. In order to determine whether particular counties were overrepresented or underrepresented among the ancestors, the geographic ancestries of 1,000 lists of random age- and sex-matched controls were traced, and empirical *p*-values were determined. As shown in Figure 2, ancestors of BARD1 variant carriers originated more often than expected from the county of S-Múlasýsla, an isolated region in the east of Iceland. An excess of ancestry, although not reaching significance, was also detected in the adjacent county A-Skaftafellssýsla. Ancestors of all the individuals tested for the BARD1 variant (irrespective of the variant carrier status identified) did not show a geographical localization to S-Múlasýsla, indicating that the overall sample collection was not biased towards individuals with ancestry from that region (data not shown). The excess of geographical ancestry of Cys557Ser carriers in S-Múlasýsla indicates that most copies of the variant now present in Iceland originated from a relatively small number of ancestors who resided in a single geographical region prior to the expansion of the Icelandic population from approximately 40,000 at the end of the 18th century to its current size of 300,000 [ 39]. The Cys557Ser variant is therefore likely to have been considerably more frequent in S-Múlasýsla than in the rest of the country five generations ago, pointing to the occurrence of a geographically localized founder effect.

We examined whether the geographical distribution of the contemporary carriers of Cys557Ser or that of their ancestors may have confounded the association between the variant and breast cancer. We performed multivariate analyses taking into account the geographical origins of the patients and controls themselves or their fifth-generation ancestors as covariates. In both cases, the relative risks of breast cancer in BARD1 variant carriers were unchanged and the significance of the findings was maintained ( *p =* 0.032 and 0.023 when adjusted for contemporary patients' geographical origins and the origins of the fifth-generation ancestors, respectively). Therefore, we conclude that the geographical distribution of the BARD1 variant, either now or in the past, does not explain its association with the disease.

### Icelandic BARD1 Cys557Ser Variants Have a Common Origin

The data from the International HapMap Project (CEU; HapMap Phase I, version 16c.1) indicated that the BARD1 gene is fully encompassed by a single linkage disequilibrium block extending approximately between coordinates 215.8 Mb and 216.0 Mb on Chromosome 2. We used a number of public domain SNPs in and near exon 6 of the BARD1 gene to search for a haplotype background (or backgrounds) of the Cys557Ser variant. The exon 6 SNPs were typed by DNA sequencing in carriers and non-carriers of the variant, including a sample of their relatives in order to establish haplotype phase. A single SNP background was identified in all carriers tested (haplotype frequency 0.55, *n =* 53) and in none of 1,197 non-carriers ( Table 4). This indicates a probable common origin for all the Icelandic BARD1 Cys557Ser variants.

**Table 4:**
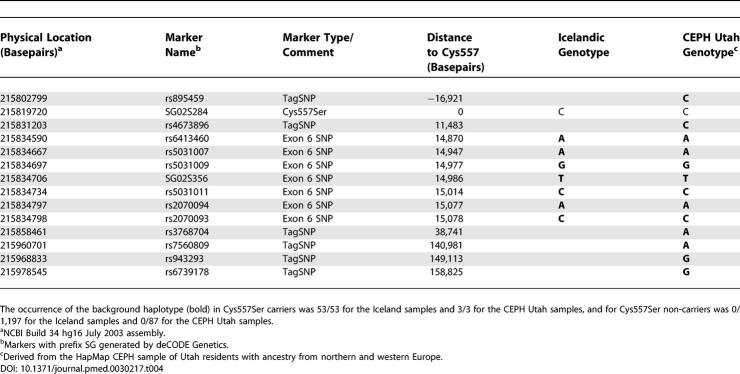
Haplotype Background of the Cys557Ser Variant

To further investigate the origins of Cys557Ser, we typed the variant in the four ethnic cohorts from the HapMap Project. The Cys557Ser variant was absent from the Han Chinese ( *n =* 45), Japanese ( *n =* 45), and Yoruba (30 triads). Three unrelated individuals in the CEPH sample of Utah residents with ancestry from northern and western Europe ( *n =* 90) were identified as carriers. These individuals shared a 176-kb haplotype of SNPs selected to tag the BARD1 linkage disequilibrium block ( Table 4). The haplotype was absent from non-carriers. In order to relate this haplotype to the Icelandic SNP haplotype, the series of BARD1 exon 6 SNPs was typed in the CEPH Utah samples. As shown in Table 4, the haplotype defined by the HapMap tagging SNPs was completely concordant with the Icelandic SNP haplotype. We concluded that the BARD1 variants present in Iceland and in the CEPH Utah samples have a common origin.

## Discussion

In this study, we report that the BARD1 Cys557Ser variant confers risk of breast cancer and that the risk extends to carriers of the Icelandic BRCA2 999del5 mutation. Carriers of the Cys557Ser variant who had breast cancer were also found to be at increased risk for developing multiple independent primary tumors.

Soon after the discovery of BARD1 as a BRCA1-interacting protein, studies were initiated to investigate a possible contribution of BARD1 variants to risk of breast cancer. Several variants were found in rare breast cancer families or in control populations; however, their contribution to the risk of disease has been uncertain [ 24, 25, 27]. Recently, the Cys557Ser variant was reported at increased frequency in hereditary breast cancer probands from Finland [ 26]. Here we confirm that the frequency of Cys557Ser is increased among patients with a high predisposition to breast cancer. We extend this observation to show that the frequency is increased in patients who were not selected for high-predisposition characteristics. We estimate an approximately 1.8-fold increase in risk conferred by the BARD1 variant, corresponding to a population attributable risk of about 2.5%. Taken in isolation, this finding does not appear to make a great contribution to potential genetic testing for breast cancer risk. However, given the current view that the residual hereditary risk of breast cancer may be characterized by extensive genetic and allelic heterogeneity [ 6, 7, 40], it is important to identify all components of the complex genetic risk. This may be a painstaking task. It has been estimated that for predisposition alleles with frequencies and risks in the range of the Cys557Ser variant, some 250–400 different genes or alleles would be required to account for the relative risk of approximately 1.8 to first-degree relatives observed for breast cancer [ 41, 42].

Karppinen et al. [ 26] reported that the frequency of the Cys557Ser variant is significantly elevated only in groups of patients with familial breast cancer. These data are similar to the initial reports for CHEK2, where the 1100delC variant was found at significantly increased frequency only in familial breast cancer patients [ 37, 43]. In our study we demonstrate significantly increased frequencies of the BARD1 variant in breast cancer patients who were not selected for family history and in probands representing high-predisposition clusters of patients. Our point estimates of ORs were slightly greater for the high-predisposition patients, although the differences were not significant (OR = 1.82, 95% CI 1.11–3.01, versus OR = 2.41, 95% CI 1.22–4.75, for high-predisposition breast cancer). It is important to consider what these combined observations imply regarding the contribution of the low-penetrance alleles to familial breast cancer. Two factors contribute to the increased prevalence of a risk allele in familial or high-predisposition patients. First, by definition, a genetic risk variant must be responsible for some familial clustering of the disease. Second, further increased familial clustering of affected carriers may result from the variant acting in concert with other predisposition determinants. Since such interactions are largely unknown or difficult to measure, it is of interest to observe directly the tendency of variant carriers to participate in familial breast cancer clusters. We show that while BARD1 Cys557Ser contributes to familial clustering of breast cancer, the effect does not exceed the innate tendency of the general population of breast cancer patients to form familial clusters. This tendency most probably reflects the uncharacterized genetic risk determinants segregating in the background breast cancer population. The practical implication of this observation is that most patients carrying the BARD1 Cys557Ser variant will present without a distinctive family history of breast cancer.

This is not to say that the BARD1 variant is trivial in familial breast cancer. We show that the risk conferred by the BARD1 Cys557Ser variant extends to BRCA2 999del5 carriers. It has been known for some time that different BRCA2-999del5-mutation-carrying families exhibit varying penetrances for breast cancer [ 12]. The BARD1 Cys557Ser variant is clearly a factor contributing to this variation. We estimate that the risk of breast cancer in a 999del5 carrier who also carries Cys557Ser is more than 3-fold greater than the risk in a 999del5 carrier who does not carry the BARD1 variant. Even though the confidence intervals on this estimate are wide (95% CI 1.16–8.40), given that BRCA2 999del5 carriers have a lifetime probability of developing breast cancer in excess of 40%, the combined risk to a Cys557Ser/999del5 double carrier could approach certainty. A positive test for Cys557Ser in a BRCA2 carrier might, therefore, have serious clinical implications.

We did not see a significant difference between the OR for Cys557Ser in BRCA2 carriers versus BRCA2 non-carriers ( Table 1). Therefore, the factor by which an individual's baseline risk is multiplied because of the presence of Cys557Ser may be the same regardless of their BRCA2 carrier status. However, since the baseline risk for BRCA2 mutation carriers is already high, the same multiplicative factor results in a very much greater increase in absolute risk in BRCA2 carriers than in BRCA2 non-carriers.

Our estimate of risk from BARD1 Cys557Ser in BRCA2 999del5 carriers assumes that the two variants are in linkage equilibrium in the population. Since the two genes are on different chromosomes, this is a reasonable assumption. All control individuals were genotyped for both the BARD1 and BRCA2 variants. This genotyping revealed no evidence to support a deviation from linkage equilibrium that might account for the increased frequencies of BARD1 Cys557Ser in BRCA2 999del5 carriers, although the power to detect a disequilibrium is limited by the low frequencies of variant carriers among the controls.

The observation of Cys557Ser risk extending to BRCA2 carriers contrasts markedly with reports of the interactions between the CHEK2 1100delC variant and BRCA mutations [ 36, 37, 43]. In the studies published to date, no CHEK2 mutation carriers have been found among BRCA mutation carriers. This underrepresentation of CHEK2 1100delC, while not statistically significant, is inconsistent with a multiplicative model of risk. It has been suggested that the paucity of BRCA mutations amongst CHEK2 1100delC carriers reflects the functional redundancy of pathways affected by BRCA and CHEK2 [ 36, 37]. It is questionable whether BARD1 and BRCA2 operate in the same biological pathways. The majority of BARD1′s biological activity is thought to be mediated through the complex with BRCA1; the interactions between BRCA1 and BRCA2 in homologous-recombination-directed DNA repair are well characterized. However, BARD1 and BRCA1 function additionally in transcription-coupled repair, where a role for BRCA2 has not been demonstrated [ 18]. BARD1 and BRCA2 pathways therefore may not overlap to the same extent as those of the CHEK2 and BRCA proteins do. The best example of overlapping pathways would of course be BARD1 and BRCA1, so it would be of great interest to investigate the risk from BARD1 Cys557Ser variants amongst BRCA1 mutation carriers. Such studies are not sufficiently well powered in Iceland because of the low frequency of carriers of known BRCA1 mutations [ 11].

The identification of individuals homozygous for BARD1 Cys557Ser demonstrates that the variant is not a recessive lethal, in contrast to observations that BARD1 knockout is lethal in mice and that knockdown mice show evidence of haploinsufficiency [ 16, 17]. This would suggest that the BARD1 Cys557Ser variant protein has residual function or that redundant pathways exist in humans. The Cys557Ser variant protein has been shown to be defective in growth suppression and the induction of apoptosis [ 31]. Further functional studies on the BARD1 protein in general and the Cys557Ser variant in particular are clearly warranted.

Lobular carcinoma is associated with familial risk of breast cancer [ 38, 44– 46]. Familial non-BRCA cancers have a higher frequency of invasive lobular carcinoma than BRCA1 cancers, suggesting that there is an uncharacterized genetic predisposition involving this tumor type [ 47]. The BARD1 Cys557Ser variant may contribute to this predisposition. There are also indications of an association between medullary cancer and familiality [ 44, 48]. Medullary and atypical medullary carcinoma have been associated with BRCA1 mutation carriers [ 49, 50]; however, this finding has not been universal [ 51– 54]. Partly, inconsistencies may arise because BRCA1 tumors exhibit certain morphological characteristics that are found in medullary carcinoma, but are not unique to this histological type [ 48]. Moreover, the association may be confounded because the largest studies used big multicancer families or groups with early-onset disease. It is possible that high-penetrance BRCA1 families may co-segregate other genetic factors that predispose to medullary-carcinoma-associated morphologies. One may speculate that the BARD1 Cys557Ser variant plays a role here, but resolution of this point would again require the identification of a sufficient number of BRCA1-mutant BARD1 Cys557Ser variant carriers.

The Cys557Ser variant has now been reported in samples from Iceland, Finland, and Italy, and in Americans of European descent, suggesting that its presence in Iceland was a result of migration rather than a de novo mutation [ 24, 26, 27]. Indeed, our finding of the same SNP haplotype among variant carriers in Iceland and in the CEPH Utah sample suggests a single ancient mutation that has become geographically widespread in European-descendant populations. In Iceland, the variant displays the characteristics of a founder effect over the last one and a half centuries. The geographical ancestry indicates that five generations ago the frequency of the variant was considerably higher in one isolated region than in the rest of the country and that most present-day copies of the variant originate from that region. The origin of this founder effect is likely to be attributable to a combination of factors including genetic drift, limited gene flow between counties, and a greater number of Cys557Ser carriers amongst the first settlers of S-Múlasýsla.

We and others did not find the BARD1 Cys557Ser variant in samples from Yoruba, Chinese, Japanese, and African-American individuals [ 25, 27]. Therefore, the variant may be restricted to individuals with European ancestry and could contribute to the higher load of breast cancer seen in this group [ 55]. However, other BARD1 variants have been discovered in African-American and Japanese individuals [ 25, 27]. The contribution of these variants to the risk of disease is still uncertain.

## Supporting Information

### Accession Numbers

The RefSeq ( http://www.ncbi.nlm.nih.gov/RefSeq) accession number for BARD1 is NM_000465 and for BRCA2 is NM_000059.

## Abbreviations

CEPH: Centre d'Etude du Polymorphisme Humain
CI: confidence interval
ICR: Icelandic Cancer Registry
OR: odds ratio
SNP: single nucleotide polymorphism
